# The Fabric of Aboriginal and Torres Strait Islander Wellbeing: A Conceptual Model

**DOI:** 10.3390/ijerph18157745

**Published:** 2021-07-21

**Authors:** Gail Garvey, Kate Anderson, Alana Gall, Tamara L. Butler, Lisa J. Whop, Brian Arley, Joan Cunningham, Michelle Dickson, Alan Cass, Julie Ratcliffe, Allison Tong, Kirsten Howard

**Affiliations:** 1 Menzies School of Health Research, Charles Darwin University, Darwin 0810, Australia; gail.garvey@menzies.edu.au (G.G.); alana.gall@menzies.edu.au (A.G.); tamara.butler@menzies.edu.au (T.L.B.); lisa.whop@anu.edu.au (L.J.W.); brian.arley@menzies.edu.au (B.A.); joan.cunningham@menzies.edu.au (J.C.); alan.cass@menzies.edu.au (A.C.); kirsten.howard@sydney.edu.au (K.H.); 2 School of Public Health, University of Queensland, Brisbane 4000, Australia; 3 Sydney School of Public Health, The University of Sydney, Sydney 2006, Australia; michelle.dickson@sydney.edu.au (M.D.); allison.tong@sydney.edu.au (A.T.); 4 National Centre for Epidemiology and Population Health, Australian National University, Canberra 2601, Australia; 5 Health and Social Care Economics Group, Caring Futures Institute, Flinders University, Adelaide 5042, Australia; julie.ratcliffe@flinders.edu.au; 6 Menzies Centre for Health Policy and Economics, The University of Sydney, Sydney 2006, Australia

**Keywords:** Aboriginal and Torres Strait Islander people, wellbeing, Indigenous, models of wellbeing, quality of life

## Abstract

Wellbeing is culturally bound and is shaped by many aspects of life, including experiences, beliefs and values. As such, in order to accurately measure wellbeing for a specific cultural group, it is necessary to understand the experiences, beliefs and values that influence the conception and experience of wellbeing of that group. This paper presents a conceptual model of wellbeing for Aboriginal and Torres Strait Islander people, which was developed from a large national qualitative study that explored the views of 359 Aboriginal and Torres Strait Islander adults. An Aboriginal- and Torres Strait Islander-led research team used an Indigenist research approach to iteratively develop this conceptual model, called the Fabric of Aboriginal and Torres Strait Islander Wellbeing model, which takes inspiration from Aboriginal and Torres Strait Islander weaving traditions whereby individual strands are twined to create fabrics that are both beautiful and strong. This reflects our findings that the parts of life that are most important to wellbeing for many Aboriginal and Torres Strait Islander people are interwoven with their families, communities and culture.

## 1. Introduction

Increasingly, research attention is being paid to understanding, defining and measuring the wellbeing of Indigenous peoples around the world [1,2,3,4]. Conceptions of wellbeing are culturally bound and shaped by a person’s experiences, beliefs and values [5]. Whilst understandings do vary across cultural groups, Indigenous peoples globally commonly hold holistic and collectivist worldviews that prioritise the wellbeing of the group above one’s own individual needs [6,7,8], differing markedly from the dominant Western individual-centric worldview. Furthermore, many Indigenous peoples share similar histories of colonisation and trauma [9]. The ongoing intergenerational legacies of colonisation, including the disruption of family and kinship networks, loss of language and culture, and social marginalisation, impact the experience and conception of wellbeing for Indigenous peoples [9].

Many parts of life influence Indigenous peoples’ wellbeing, and these parts are often described by Indigenous people, globally, as being deeply and inextricably interconnected [8,10]. For example, Kilcullen et al. (2016) identified connectedness to country, family and kinship, cultural knowledge and social networks as contributing to the health and wellbeing of Aboriginal and Torres Strait Islander people in Australia [11]. The collective importance of family (including those beyond immediate blood relations) and kinship to Indigenous people’s wellbeing is a recurring theme that informs many wellbeing frameworks for Indigenous people globally [2,12,13,14,15,16,17,18]. Butler et al. (2019) identified several interconnected components that contribute to Aboriginal and Torres Strait Islander peoples’ wellbeing, including: autonomy, empowerment and recognition; family and community; culture, spirituality and identity; country; basic needs; work, roles and responsibilities; education; and physical and mental health [10].

Despite the significant literature supporting the importance of the interconnection of dimensions of wellbeing for Aboriginal and Torres Strait Islander people, existing measures [8,10] and indicators do not adequately capture these holistic and collectivist aspects. For example, while successive Australian governments have committed to improving the health and wellbeing of Aboriginal and Torres Strait Islander people, to date, wellbeing has not been captured using measures that are based on the values and preferences of Aboriginal and Torres Strait Islander people [4,19]. Non-Indigenous quality of life measures, adapted clinical measures or generic ‘indicators’ (e.g., income, level of education, household size) have instead been used to measure quality of life and wellbeing [20,21,22]. Existing quality of life tools are underpinned by Western biomedical constructs of wellbeing and quality of life that focus on an individual’s health and absence of disease [23]. They cover topics such as physical state (mobility, sensation, vision, hearing), psychological state (happiness, depression, anxiety, self-worth, coping), pain, self-care, usual activities and relationships. Easily measurable ‘indicators’ of wellbeing, such as level of education [24], tend to be considered in isolation as markers or predictors of wellbeing without fully considering the context in which they sit. Considering these ‘indicators’ of wellbeing in isolation ignores the importance of the interconnectedness between the different components of wellbeing for Aboriginal and Torres Strait Islander people, and, as a result, their use will not accurately reflect a person’s overall wellbeing [2]. The absence of a robust wellbeing measure has significantly hindered the ability to monitor and evaluate progress in improving the wellbeing of Aboriginal and Torres Strait Islander people [25,26].

Our companion paper [27] in this Special Issue describes the foundations of wellbeing identified and valued by Aboriginal and Torres Strait Islander adults as important to their overall wellbeing and having a good life. These aspects of wellbeing will inform the development of a nationally relevant wellbeing measure for Aboriginal and Torres Strait Islander adults. An important issue that remains to be addressed is how the domains of wellbeing for Aboriginal and Torres Strait Islander people, and the connections between them, can be theoretically conceptualised. Thus, the aim of this paper is to present a new conceptual model of Aboriginal and Torres Strait Islander adult wellbeing and describe the interrelationship of the dimensions identified as important to wellbeing.

## 2. Materials and Methods

This paper presents the findings from one component of the qualitative phase of a larger mixed-methods study titled ‘What Matters 2 Aboriginal and Torres Strait Islander Adults’ (WM2Adults). WM2Adults aims to develop a nationally relevant, strengths-based measure of wellbeing that is grounded in the parts of life that are important to Aboriginal and Torres Strait Islander adults. Such a measure will increase the accuracy and acceptability of wellbeing measurement and valuation for Aboriginal and Torres Strait Islander adults to more effectively inform decision making to improve the health and wellbeing of this population. The overall WM2Adults project methods are described elsewhere [28], and the methods for the qualitative phase are described in a companion article titled ‘What Matters 2 Adults (WM2Adults): Understanding the Foundations of Aboriginal and Torres Strait Islander Wellbeing’ in this Special Issue [27]. The focus of the current paper is the development of a new conceptual model of wellbeing for Aboriginal and Torres Strait Islander adults, which was borne out of the qualitative phase of the WM2Adults study. As such, the methods described in detail here relate specifically to the research processes undertaken by our team in developing the conceptual model.

### 2.1. Research Team

Our team acknowledges the importance of reflexively considering and describing our own backgrounds, perspectives and values that we each bring to the project [29,30]. The first author (GG) is a senior Aboriginal researcher with extensive research experience in Aboriginal health, and members of our team include Aboriginal and Torres Strait Islander junior and mid-career researchers (AG, TB, LJW, BA, MD). Our team also brings experience and expertise in Aboriginal and Torres Strait Islander health research, including qualitative research (GG, KA, AG, TB, MD, AT), outcomes measurement, health economics and preferences (KH, JR) and epidemiology (AC, JC, LJW).

### 2.2. Methodology

The methodology used in the WM2Adults study is underpinned by an Indigenist research approach, which is informed by three core principles: (1) notions of resistance as part of Aboriginal and Torres Strait Islander peoples’ continued fight for self-determination; (2) leadership of Aboriginal and Torres Strait Islander researchers in representing their communities to achieve self-determination; and (3) ensuring political integrity and privileging voices of Aboriginal and Torres Strait Islander people [31]. Supporting this approach has been the use of culturally appropriate research methods for gathering, interpreting and sharing the stories and ideas of Aboriginal and Torres Strait Islander people. The use of the yarning research process [32] is central to our methodology. Yarning is a method of gathering information that respects Aboriginal and Torres Strait Islander peoples’ oral traditions and values, and the method privileges Aboriginal and Torres Strait Islander knowledge in a culturally safe manner [32,33,34].

### 2.3. Participants and Data Collection

Aboriginal and Torres Strait Islander adults (18 years of age or older at the time of recruitment) were purposively recruited from a broad range of participating community organisations (e.g., community art and cultural centres and health services) and groups (e.g., men’s and women’s, Elders’ and sporting and recreational groups). A local contact at each of the 20 participating sites assisted the research team with recruiting participants and organising a suitable venue, time and day to hold the yarning circles. Yarning circles [32] were used to gather data from Aboriginal and Torres Strait Islander adults about wellbeing between September 2017 and September 2018 across a diverse range of geographical regions across Australia, which is reported in more detail in our companion paper [27].

### 2.4. Ethics Approval

Formal ethics approvals were obtained from the Human Research Ethics Committee of the Northern Territory Department of Health and Menzies School of Health Research (Ref: 2017–2855 and Ref. 2019–3333); University of Sydney Human Research Ethics Committee (Ref: 2017/724 and Ref. 2019/672); Central Australian Aboriginal Congress Aboriginal Corporation; Central Australian Human Research Ethics Committee; Western Australian Aboriginal Health Ethics Committee (Ref: 833); Aboriginal Health & Medical Research Council (Ref: 1340/17); Aboriginal Health Council of South Australia’s Aboriginal Health Research Ethics Committee (Ref: 04-17-741); St Vincent’s Hospital Melbourne Human Research Ethics Committee (Ref: 034/18); and the Charles Darwin University Human Research Ethics Committee (Ref: H19059).

### 2.5. Data Analysis

**Figure 1:**
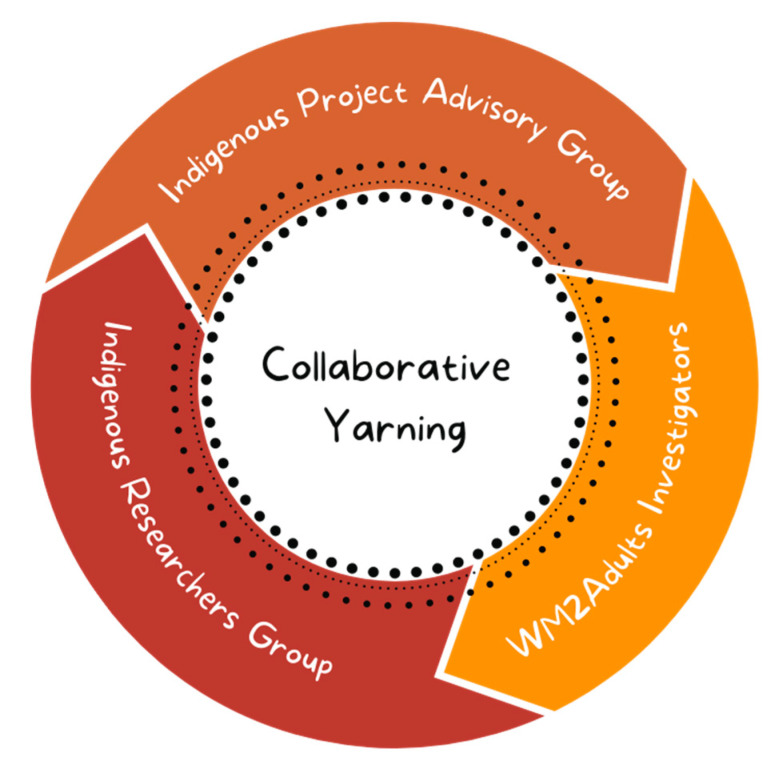
Groups involved in the collaborative yarning process.

Consistent with the yarning research process [32], an extensive and iterative process of collaborative yarning [35] was used to analyse and interpret the data collected via the yarning circles. Collaborative yarning is a culturally appropriate method of analysis that uses a flexible and inclusive approach to allow multiple researchers and stakeholders to be more engaged with the process of the research and co-analysis of the data [32,35]. Three groups were involved in the collaborative yarning process—the WM2Adults investigators, an Indigenous Project Advisory Group (IPAG) and a purposively convened group of Aboriginal and Torres Strait Islander researchers (Indigenous Researchers Group—IRG). The IRG was led by the WM2Adults Senior Aboriginal Researcher (GG) and comprised Aboriginal and Torres Strait Islander study investigators and research staff, who provided additional in-depth guidance with the data analysis and interpretation (see Figure 1). Two people were members of both the IRG and the WM2Adults investigators (GG and LJW), and one person was a member of the WM2Adults investigators and IPAG (MD).

**Figure 2:**
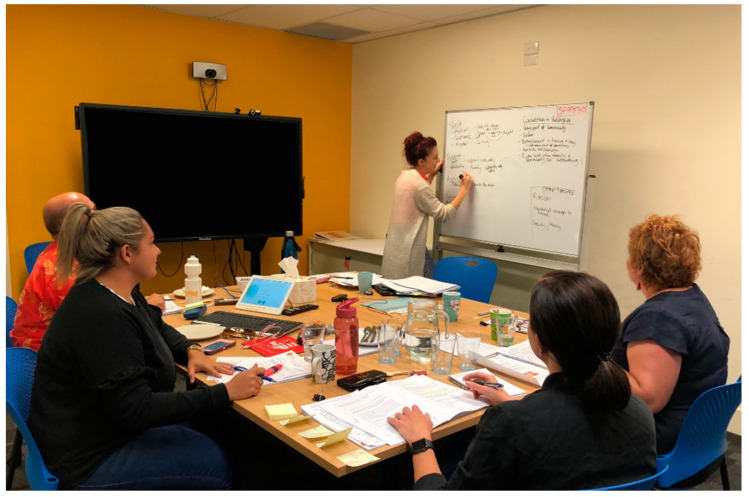
Meeting of the Indigenous Researchers Group (IRG).

An adapted grounded theory approach and reflexive thematic analysis [36] were used whereby two researchers (KA—an experienced non-Indigenous qualitative researcher, and TB—an Aboriginal early career qualitative researcher) initially reviewed a sample of the transcripts line-by-line to inductively generate a draft thematic framework relating to the parts of life identified as important to the wellbeing of participants. Emerging themes, with collated supporting raw data, were presented to and discussed by the IRG during three half-day yarning sessions over a six-week period during 2018 (see Figure 2). During these sessions, the IRG considered and discussed the assumptions and interpretations apparent in the emerging themes, their reflections on the themes and how the themes fit together. GG then reported the findings of the IRG meeting back to the WM2Adults investigators to guide and adapt the ongoing process of reflexive thematic analysis.

During the collaborative and iterative process of review and refinement from the IRG, a conceptual model of wellbeing began to develop through the discussions between IRG members and the WM2Adults investigators. During the third meeting of the IRG, a draft conceptual model of wellbeing, called the Fabric of Aboriginal and Torres Strait Islander Wellbeing, was proposed.

This draft model was presented at the face-to-face meeting of the IPAG in Darwin in February 2019, which led to further refinement of the descriptions of the aspects of wellbeing and endorsement of the new conceptual model. The IPAG also provided guidance on the wording and conceptualisation of the themes.

## 3. Results

### 3.1. Profile of Participants

As described in our companion paper [27], 359 Aboriginal and Torres Strait Islander adults participated in this study. In our study, 57.1% of participants were female, the median age of participants was 50 years, the ages ranged from 18 to 92 years, 84.4% were Aboriginal, 5.8% were Torres Strait Islander, 6.4% were both Aboriginal and Torres Strait Islander, 80.5% spoke English as their main language at home, 57.1% resided in regional areas and 53.9% had an educational attainment level equivalent to grade 10 or below.

### 3.2. The Fabric of Aboriginal and Torres Strait Islander Wellbeing Model

Following an extensive process of collaborative yarning between the WM2Adults investigators, the IRG and IPAG, the new model, called the Fabric of Aboriginal and Torres Strait Islander Wellbeing, was finalised (see Figure 2). The nature and foundations of this model are presented and described here in detail, with supporting quotes from yarning circle participants, to illustrate particular aspects of the model.

The most essential characteristic of wellbeing for Aboriginal and Torres Strait Islander people, as reported by the participants, and further reinforced by the IRG and IPAG members, is its deeply interwoven and collectivist nature. It was evident that while discrete aspects of life that impact on wellbeing can be identified, it is the way in which these aspects fit together that is regarded as encapsulating the essence of a person’s overall sense of wellbeing.

*‘[Different parts of life] are all connected to make one… But even the healthy home life and family, you know, my connection to community or country. So, you can’t put these in different categories. So, that’s just one category… All of those make up me.’*

(Participant, Tasmania, Outer Regional)

This interwoven understanding of wellbeing was commonly expressed by participants as the tethering aspects of an individual’s wellbeing to the impact on the individual’s family, community and culture. The importance of all parts of life to the participants was not just the impact on the individual, but on the collective contexts and connections. In this way, it was understood that for an individual’s wellbeing to be strong, the impact of the critical parts of life on the individuals’ family, community and culture needed to be positive and supportive of the collective.

*‘When you ask an individual [about their wellbeing], they will not tell you about their own individual stresses or how they are. As long as their family is okay, then everything is okay.’*

(Participant, Northern Territory, Major City)

The process of collaborative yarning identified three fundamental connective threads that run through an individual’s wellbeing: family, community and culture. These three connective threads were understood as inextricably interwoven in and around the critical parts of life that impact the wellbeing of Aboriginal and Torres Strait Islander people: belonging and connection; holistic health; purpose and control; dignity and respect; and basic needs. The qualitative analysis of the content of each thread is presented in-depth in our companion paper in this Special Issue [27]. Briefly, belonging and connection was the theme most commonly spoken about by participants; it was predominantly associated with an individual’s relationships and bonds with family and community, and the importance of culture in developing and maintaining a sense of shared experience and understanding. Holistic health referenced the idea that being healthy was an essential component of wellbeing; health was described as a holistic and multidimensional state of wellness often determined the quality of one’s connections to family, community and culture. Purpose and control were fundamental to wellbeing and were most often discussed in the context of stability at home, employment and financial security, education and cultural and familial responsibilities. The importance to wellbeing of an individual feeling that they were viewed and treated by others with dignity and respect was described, which was experienced in the contexts of interpersonal relationships, policies, services and experiences of racism. Participants also expressed the imperative to have their needs, and the needs of their family and community met, in order to move forward and achieve good wellbeing. Basic needs identified by participants included: housing, money, access to services, education, employment, opportunities to thrive and justice.

**Figure 3:**
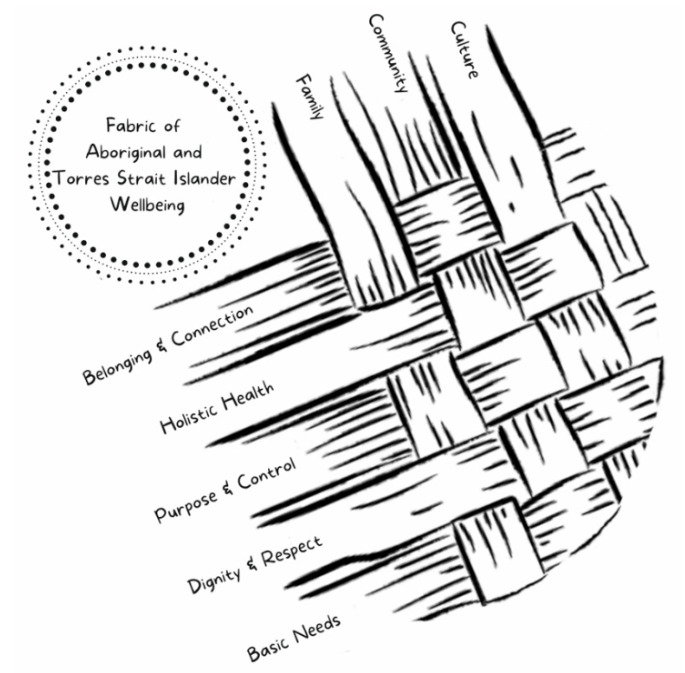
The Fabric of Aboriginal and Torres Strait Islander Wellbeing conceptual model.

The distinctive characteristic of these interwoven parts of life being so central to the way in which wellbeing is understood and experienced by Aboriginal and Torres Strait Islander people led to our team’s conceptualisation of the Fabric of Aboriginal and Torres Strait Islander Wellbeing model (see Figure 3).

**Figure 4:**
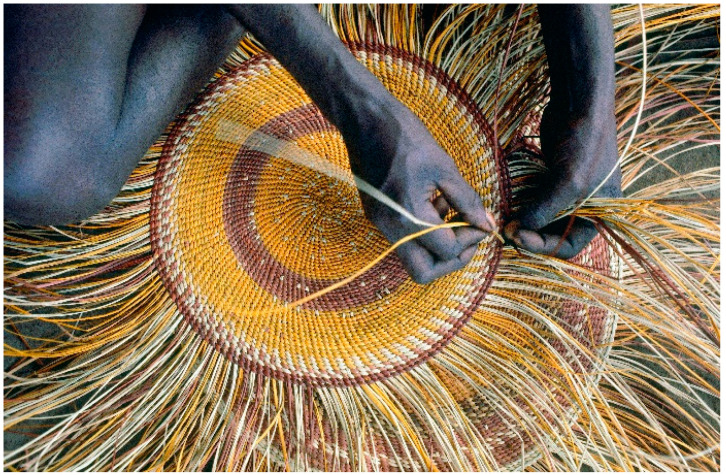
Traditional weaving in the Tiwi Islands. Photo by Penny Tweedie, used with permission.

This model takes inspiration from Aboriginal and Torres Strait Islander peoples’ weaving traditions (see Figure 4). In the way that weavers take the individual strands of reeds or leaves and twine them together to create fabrics, baskets and fishing nets that are both beautiful and strong, the parts of life that are most important to wellbeing for many Aboriginal and Torres Strait Islander people are interwoven through their families, communities and culture.

*‘Because Country and culture is connected and spiritual connection, passing on of knowledge is connected to country and culture… And your identity… And family… [and] …education.’*

(Participant, Tasmania, Major City)

This model represents the characteristic that the strength of wellbeing is derived from both the strength of the threads and their connections with each other. Thus, the strength of a person’s overall wellbeing can be understood as the strength of the connection between these interwoven threads. Moreover, it was articulated that each of the threads and connections hold importance to a person’s wellbeing.

*‘I don’t think you can put these [parts of life] in any order… It’s all interconnected.’*

(Participant, New South Wales, Inner Regional)

Participants commonly felt that their deeply interwoven and connected conception of wellbeing is rarely, if ever, recognised or acknowledged by governments and institutions that provide services and programs to Aboriginal and Torres Strait Islander people. This was regarded as a major impediment to the success of policies and programs that are intended to improve the wellbeing of Aboriginal and Torres Strait Islander people.

*‘I think the mistake that’s made too often is compartmentalising things and someone said it’s all important and it is all important… if we try to put things into boxes—like white people do, that’s where we go wrong with our policies.’*

(Participant, Tasmania, Major City)

The *Fabric of Aboriginal and Torres Strait Islander Wellbeing* model is a representation of the understanding that it is the strength of one’s connections and of achieving balance in all things that is the key to having a good wellbeing.

*‘So you need to make [wellbeing measurement] more holistic, taking into account the balance every time.’*

(Participant, Tasmania, Major City)

The companion paper in this Special Issue [27] provides an in-depth exploration and additional examples of the deeply interconnected nature of wellbeing for Aboriginal and Torres Strait Islander people.

## 4. Discussion

This paper presents the process and results of our analysis that developed a new conceptual model for Aboriginal and Torres Strait Islander wellbeing—a conceptualisation that focuses on the interwoven nature of wellbeing aspects, and the idea that strong wellbeing comes from the strength and balance of the threads of the Fabric of Aboriginal and Torres Strait Islander Wellbeing. The Fabric of Aboriginal and Torres Strait Islander Wellbeing conceptual model acknowledges the importance of the discrete threads of one’s wellbeing but goes further to the importance of the relationship between these. It also recognises that the parts of life that are most important to wellbeing for many Aboriginal and Torres Strait Islander people are interwoven through their families, communities and culture, in the same way that weavers take individual strands and weave them together to make fabrics, such as baskets and fishing nets, that are extremely strong—stronger than any of the individual threads in isolation. This model echoes the participants’ strong views that one’s wellbeing is deeply interwoven and interconnected.

The Fabric of Aboriginal and Torres Strait Islander Wellbeing conceptual model also draws on the strengths of the extensive work conducted in Australia over many years on the social and emotional wellbeing (SEWB) of Aboriginal and Torres Strait Islander people [6,37,38]. Existing SEWB models include connections to land, culture and spirituality and recognise mental wellbeing as an important component that is inextricably linked to the social, emotional, physical, cultural and spiritual dimensions of wellbeing [6,38]. SEWB incorporates an ecological, collectivist perspective; this is also consistent with our findings that wellbeing is intrinsically embedded within family, culture and community [27]. This requires wellbeing to be considered within a framework that encompasses a holistic worldview, as described below.

*‘Aboriginal health does not mean the physical wellbeing of an individual, but refers to the social, emotional, and cultural wellbeing of the whole community. For Aboriginal people this is seen in terms of the whole-life-view. Health care services should strive to achieve the state where every individual is able to achieve their full potential as human beings and must bring about the total wellbeing of their communities.’*

[6] (p. 56)

In seeking to improve wellbeing for Aboriginal and Torres Strait Islander people, the limitations of existing Western biomedical constructs of wellbeing, which focus on the individual, must be recognised [23]. Measures of wellbeing must also capture Aboriginal and Torres Strait Islander peoples’ holistic worldviews and the collective and interconnected conceptualisation of different aspects of wellbeing. Instruments should be valid and robust and include domains of wellbeing that are most relevant to Aboriginal and Torres Strait Islander people [26]. Such measures should capture an individual’s perceptions of wellbeing, as well as conceptual notions of wellbeing in the context of their culture and value systems [39]. Existing measures of quality of life that are often employed as pseudo-‘wellbeing’ measures by health services and policy makers simply do not achieve this—they almost exclusively focus on dimensions of health and health status, without including dimensions of wellbeing, nor do they consider the social and cultural context in which Aboriginal and Torres Strait Islander people may live, and their ways of being [40]. This new Fabric of Aboriginal and Torres Strait Islander Wellbeing conceptual model goes beyond the Western notions of health and wellbeing and their focus on the individual.

There is a growing interest, both in Australia and globally, in measuring and quantifying Indigenous peoples’ wellbeing using approaches that acknowledge and value their cultures and ways of knowing, being and doing [41]. In Australia, a number of studies have been conducted largely within discrete Aboriginal and Torres Strait Islander communities, settings or populations, and these have identified some important aspects of wellbeing [1,13,42,43]. No measures that are routinely used by health services and policy makers consider the interconnection of dimensions or are informed by the voices, values and preferences of Aboriginal and Torres Strait Islander people.

### Strengths and Limitations

The strength of our study lies in our methods and approach to ensure that Aboriginal and Torres Strait Islander voices were privileged at every step of the data collection, analysis and model development stages. We constantly referred to and gave priority to the words and concepts that came directly from the participants in our yarning circles. Through a process of collaborative yarning between the WM2Adults investigators, the Indigenous Project Advisory Group and the Indigenous Researchers Group, we reached a collective understanding that the strength of overall wellbeing comes from the connections between different aspects of wellbeing. Our study team is led by a senior Aboriginal researcher with extensive research experience in Aboriginal health, and members of our team also included Aboriginal and Torres Strait Islander junior and mid-career researchers, giving a range and depth of research experiences, as well as the strength of our individual and collective lived experiences as Aboriginal and Torres Strait Islander people, on which to draw. One limitation of our study was that we were not able to present this new conceptual model back to all of the yarning circle participants due to the resource and time limitations; however, we did include the model in our study newsletter which was emailed to those participants who indicated they would like to receive study updates. This new conceptual model was presented to and endorsed by the IPAG.

## 5. Conclusions

This paper contributes to our understanding of wellbeing from an Aboriginal and Torres Strait Islander perspective. This new Fabric of Aboriginal and Torres Strait Islander Wellbeing conceptual model will be used to inform the development of a holistic measure of wellbeing that can be used by health services and policy makers working with and for Aboriginal and Torres Strait Islander people.

## Data Availability

Data may be available from authors on request.
